# Optimal timing and dosage of chemotherapy as a combined treatment with androgen withdrawal in the human prostate LNCaP tumour model

**DOI:** 10.1054/bjoc.2000.1686

**Published:** 2001-03

**Authors:** H Miyake, S Hara, S Arakawa, S Kamidono, I Hara

**Affiliations:** Department of Urology, Kobe University School of Medicine, 7-5-1 Kusunoki-cho, Chuo-ku, Kobe, 650-0017, Japan

**Keywords:** prostate cancer, mitoxantrone, castration, androgen-independent progression, apoptosis

## Abstract

Although numerous chemotherapeutic agents have been evaluated for patients with advanced prostate cancer, none have demonstrated improved survival benefits. Here, in order to determine whether the efficacy of chemotherapy can be enhanced by changing the regimen, we evaluated the effect of the varied timing and dosage of chemotherapy in combination with androgen withdrawal on time to androgen-independent (AI) progression in the human androgen-dependent LNCaP tumour model. We first demonstrated the synergistic effect of mitoxantrone on induction of apoptosis in LNCaP cells maintained in the steroid hormone-depleted charcoal-stripped media (CSM) compared to those in the standard media. In addition, this synergy was most remarkable during the simultaneous treatment of LNCaP cells with mitoxantrone and CSM compared to the pre- or post-use of mitoxantrone with CSM. LNCaP tumour growth in athymic nude mice and their increase in serum PSA levels were significantly inhibited by the simultaneous injection of mitoxantrone with castration, compared to the administration of mitoxantrone 2 weeks before or after castration. The TUNEL staining revealed that apoptotic cell death was extensively induced in LNCaP tumours treated with simultaneous castration and mitoxantrone compared to tumours treated with the other schedules. Furthermore, nude mice bearing LNCaP tumours were injected with a total of 0.5 mg mitoxantrone divided into 2, 5 and 10 days, with the most significant therapeutic effect and delayed AI progression observed in mice given injection of mitoxantrone for 2 days. These findings suggest that this might be the optimal way to perform cytotoxic chemotherapy and androgen withdrawal simultaneously in patients with advanced prostate cancer and to administer chemotherapeutic agents at high dosage within short intervals. © 2001 Cancer Research Campaign http://www.bjcancer.com


British Journal of Cancer (2001) **84**(6), 859–863
© 2001 Cancer Research Campaign
doi: 10.1054/ bjoc.2000.1686, available online at http://www.idealibrary.com on IDEAL®

http://www.bjcancer.com

### Optimal timing and dosage of chemotherapy as a combined treatment with androgen withdrawal in the human prostate LNCaP tumour model

**H Miyake, S Hara, S Arakawa, S Kamidono and I Hara**

Department of Urology, Kobe University School of Medicine, 7-5-1 Kusunoki-cho, Chuo-ku, Kobe 650-0017, Japan

**Summary** Although numerous chemotherapeutic agents have been evaluated for patients with advanced prostate cancer, none have demonstrated improved survival benefits. Here, in order to determine whether the efficacy of chemotherapy can be enhanced by changing the regimen, we evaluated the effect of the varied timing and dosage of chemotherapy in combination with androgen withdrawal on time to androgen-independent (AI) progression in the human androgen-dependent LNCaP tumour model. We first demonstrated the synergistic effect of mitoxantrone on induction of apoptosis in LNCaP cells maintained in the steroid hormone-depleted charcoal-stripped media (CSM) compared to those in the standard media. In addition, this synergy was most remarkable during the simultaneous treatment of LNCaP cells with mitoxantrone and CSM compared to the pre- or post-use of mitoxantrone with CSM. LNCaP tumour growth in athymic nude mice and their increase in serum PSA levels were significantly inhibited by the simultaneous injection of mitoxantrone with castration, compared to the administration of mitoxantrone 2 weeks before or after castration. The TUNEL staining revealed that apoptotic cell death was extensively induced in LNCaP tumours treated with simultaneous castration and mitoxantrone compared to tumours treated with the other schedules. Furthermore, nude mice bearing LNCaP tumours were injected with a total of 0.5 mg mitoxantrone divided into 2, 5 and 10 days, with the most significant therapeutic effect and delayed AI progression observed in mice given injection of mitoxantrone for 2 days. These findings suggest that this might be the optimal way to perform cytotoxic chemotherapy and androgen withdrawal simultaneously in patients with advanced prostate cancer and to administer chemotherapeutic agents at high dosage within short intervals. © 2001 Cancer Research Campaign http://www.bjcancer.com

**Keywords**: prostate cancer; mitoxantrone; castration; androgen-independent progression; apoptosis

Prostate cancer is the most frequently diagnosed malignancy and the second leading cause of cancer-related deaths of men in Western industrialized countries. Androgen withdrawal remains the only effective form of systemic therapy for men with advanced disease, with symptomatic and/or objective response in 80% of the patients. Progression to androgen-independence, however, occurs within a few years in the majority of these cases (Denis and Murphy, 1993). Despite several hundred clinical studies of both experimental and approved chemotherapeutic agents, chemotherapy has limited antitumour activity with an objective response rate of less than 10% and no demonstrated survival benefit in advanced disease (Oh and Kantoff, 1998). Therefore, androgen-independent (AI) disease is the main obstacle to improving the survival and quality of life in patients with advanced prostate cancer, and the means to enhance the efficacy of cytotoxic chemotherapy for patients with advanced disease is required.

Progression to androgen-independence is a complex process including various combinations of clonal selection, adaptive up-regulation of antiapoptotic genes, androgen receptor transactivation in the absence of androgen or increased levels of co-activators and alternative growth factor pathway (Tang and Porter, 1997). The association between these mechanisms and chemoresistant phenotype of prostate cancer remains unclear. Furthermore, although recent phase II reports using chemotherapeutic agents are documenting improved response rates in hormone-refractory disease (Hudes et al, 1995; Tannock et al, 1996; Kantoff et al, 1999), it has not been well analysed whether the efficacy of such agents is enhanced, if they are administered earlier to achieve synergistic or additive effects with androgen withdrawal.

To date, controlled study of the complex process associated with progression to androgen-independence has been difficult due to a lack of an ideal animal model that mimics the clinical course of the disease in men. Of the available human prostate cancer cell lines, only the LNCaP cell line is androgen-dependent (AD), prostate-specific antigen (PSA) secreting and immortalized in vitro. As in human prostate cancer, serum PSA levels in the LNCaP tumour model are initially regulated by androgen, directly proportional to tumour volume, and increase after prolonged periods following castration to progression to androgen-independence. Apoptotic tumour regression does not consistently occur after castration, but tumour growth is inhibited. Moreover, the serum PSA levels decrease by 80% for several weeks after castration, after which LNCaP tumour growth rates increase with the loss of androgen-regulated PSA gene expression as a surrogate end-point of AI progression (Gleave et al, 1999; Miyake et al, 2000a). Therefore, this model is particularly useful to test the efficacy of agents targeting castration-induced apoptosis and their effects on time to AI progression.

In this study, we first characterized the effects of combined androgen ablation and cytotoxic chemotherapy on the apoptotic features in LNCaP cells. Subsequently, the effect of the varied

*Received 17 August 2000*
*Revised 13 December 2000*
*Accepted 15 December 2000*

*Correspondence to:* H Miyake timing and dosage of chemotherapy in combination with castration on time to AI progression in the LNCaP tumour model was evaluated in order to determine whether the efficacy of chemotherapy can be enhanced by changing its regimen.

### MATERIALS AND METHODS

### Tumour cell line

LNCaP cells were kindly provided by Dr LWK Chung (University of Virginia, Charlottesville, Virginia, USA) and maintained in RPMI 1640 (Life Technologies Inc, Gaithersburg) supplemented with 5% heat-inactivated fetal calf serum. Steroid hormone-depleted charcoal-stripped media (CSM) was prepared as described previously (Miyake et al, 2000b).

### Chemotherapeutic agent

Mitoxantrone was purchased from Wyeth-Ayerst Inc (Montreal, Canada). Stock solutions of mitoxantrone (1 mg $m^{-1}$) were prepared with dimethyl sulfoxide (DMSO), and diluted with PBS to the required concentrations prior to each in vitro experiment.

### In vitro cell growth assays

The in vitro growth of LNCaP cells were assessed by the in vitro mitogenic assay as described previously (Miyake et al, 2000b). Briefly, $3 \times 10^3$ cells were seeded in each well of 96-well microtitre plates and allowed to attach overnight. After treatment with mitoxantrone, LNCaP cells were fixed with 1% glutaraldehyde (Sigma Chemical Co, St Louis) and stained with 0.5% crystal violet (Sigma Chemical Co) The optical density was determined with a microculture plate reader (Becton Dickinson Labware, Lincoln Park) at 540 nm. Absorbance values were normalized to the values obtained for the vehicle-treated cells to determine the percent of survival. Each assay was performed in triplicate.

### DNA fragmentation analysis

The nucleosomal DNA degradation was analysed as described previously with a minor modification (Miyake et al, 2000c). Briefly, $1 \times 10^5$ cultured cells were seeded in 5-cm culture dishes and allowed to adhere overnight. After indicated treatment with 0.01 μM mitoxantrone, cells were harvested and then lysed in a solution containing 100 mM NaCl, 10 mM Tris pH 7.4, 25 mM EDTA, and 0.5% SDS. After centrifugation, the supernatants were incubated with 300 μg $ml^{-1}$ proteinase K for 5 h at 65˚C and extracted with phenol-chloroform. The aqueous layer was treated with 0.1 volume of 3 M sodium acetate, and the DNA was precipitated with 2.5 volumes of 95% ethanol. Following treatment with 100 μg $ml^{-1}$ RNase A for 1 h at 37˚C, the sample was electrophoresed on a 2% agarose gel and stained with ethidium bromide.

### Assessment of in vivo LNCaP tumour growth and determination of serum PSA levels

One million LNCaP cells were inoculated subcutaneously with 0.1 ml of Matrigel (Becton Dickinson Labware) in the flank region of 6- to 8-week-old male athymic nude mice (BALB/c strain; CLEA Japan Inc, Tokyo). In the first set of experiments, 0.05 mg mitoxantrone was administered once daily by intravenous injection into each nude mouse for 5 continuous days. When tumours reached about 200 $mm^3$ in volume, mice were randomly selected for administration of mitoxantrone from 2 weeks before castration (group 1), the day of castration (group 2), 2 weeks after castration (group 3) or no treatment with mitoxantrone as the control (group 4). In the second set of experiments, a total of 0.5 mg mitoxantrone was administered by intravenous injection into each nude mouse. When tumours reached about 200 $mm^3$ in volume, mice were castrated and randomly selected for administration of 0.25 mg mitoxantrone from the day of castration to 1 day after castration (group 1), 0.1 mg from the day of castration to 4 days after castration (group 2), or 0.05 mg from the day of castration to 9 days after castration (group 3). Each experimental group consisted of eight mice.

Mice were castrated via a scrotal approach, and tumour volume was measured once weekly and calculated by the formula length × width × depth × 0.5236 as described previously (Miyake et al, 2000a). Blood samples were obtained with tail-vein incisions of mice once weekly. Serum PSA levels were determined by an enzymatic immunoassay kit with a lower limit of sensitivity of 0.2 μg $l^{-1}$ (Abbott IMX, Montreal) according to the manufacturer's protocol. Data points were reported as mean values with standard deviation.

### TUNEL staining

A modified TUNEL technique was used to detect apoptotic cells in LNCaP tumours using the ApopTag In situ Apoptosis Detection System (Oncor, Gaithersburg) according to the manufacture's protocol. The number of positively stained cells per high-power field in five random fields was counted and averaged.

### RESULTS

### Synergistic effect of mitoxantrone and androgen ablation on induction of apoptosis in LNCaP cells

To determine whether androgen ablation enhances the cytotoxic effect of mitoxantrone on LNCaP cells, we treated LNCaP cells with various doses of mitoxantrone maintained in standard medium or CSM, and the in vitro mitogenic assays were performed to evaluate the cell viability. As shown in Figure 1A, androgen ablation with the use of CSM-enhanced mitoxantrone chemosensitivity in a dose-dependent manner, reducing the $IC_{50}$ of mitoxantrone by more than 50%.

The DNA fragmentation assay was performed to analyse the effect of treatment with sublethal dose of mitoxantrone maintained in standard medium or CSM on induction of apoptotic cell death. The characteristic apoptotic DNA ladder was observed only after treatment with mitoxantrone in CSM (Figure 1B).

### Cytotoxic effects of mitoxantrone on LNCaP cells according to the timing of androgen ablation

To assess the optimal timing of chemotherapy, the in vitro mitogenic assays were used to examine the viabilities of LNCaP cells at the pre-, simultaneous- or post-use of mitoxantrone with CSM. As shown in Figure 2, simultaneous use of LNCaP cells with mitoxantrone and CSM achieved the most remarkable growth-inhibitory effect among these three treatment schedules;

 © 2001 Cancer Research Campaign

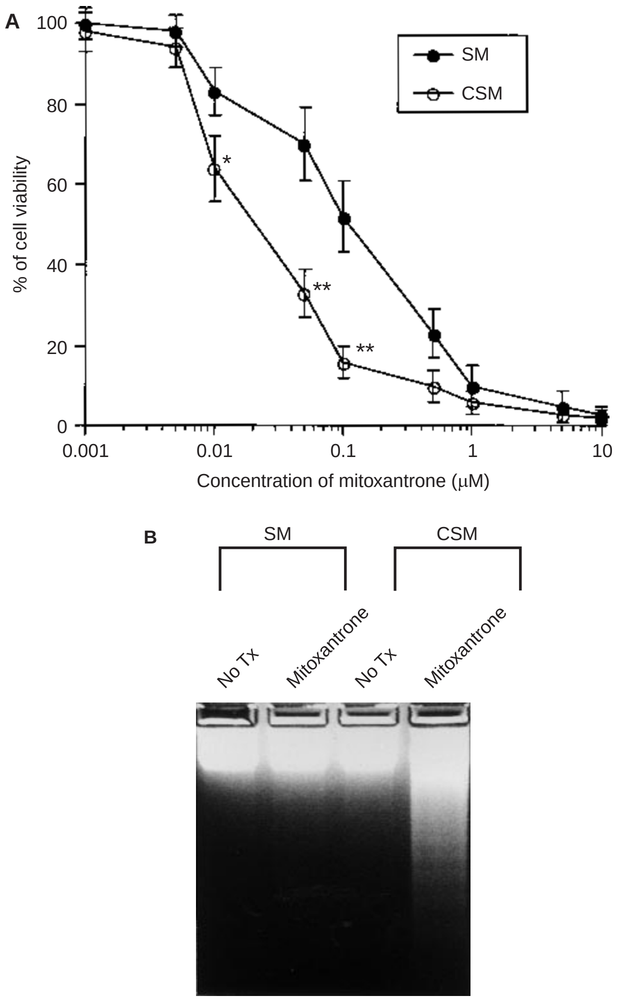

**Figure 1** (**A**) Effect of mitoxantrone on proliferation of LNCaP cells maintained in standard media (SM) or charcoal-stripped media (CSM). Cell viability was determined by the in vitro cell growth assay. Each point represents the mean of triplicate analyses with standard deviation. ** and * differ from the cells maintained in SM ($P < 0.01$ and $P < 0.05$, respectively) by Student's *t*-test. (**B**) DNA fragmentation analysis of LNCaP cells maintained in SM or CSM after treatment with mitoxantrone that is, simultaneous androgen ablation with CSM reduced the $IC_{50}$ of mitoxantrone by approximately 50% compared to other treatment schedules.

### Effects of mitoxantrone therapy on LNCaP tumour growth according to the timing of castration

We then evaluated the efficacy of mitoxantrone treatment for inhibiting the growth of subcutaneous LNCaP tumours according to the timing of castration. Male mice bearing LNCaP tumours were randomly selected for mitoxantrone treatment from 2 weeks before castration (group 1), the day of castration (group 2), 2 weeks after castration (group 3) or no treatment with mitoxantrone (group 4). Figures 3A and 3B illustrate the changes in mean LNCaP tumour volumes and serum PSA levels, respectively.

Consistent with our in vitro studies, mitoxantrone achieved the most significant inhibition of the LNCaP tumour growth, when administered simultaneously with castration. By 12 weeks after castration, the tumour volumes in groups 1, 3 and 4 increased

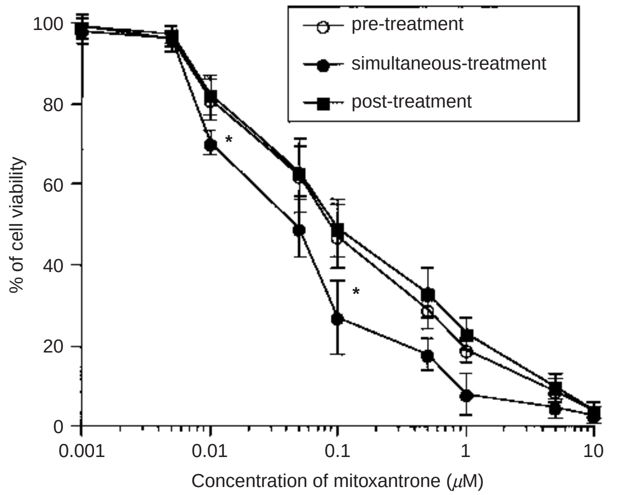

**Figure 2** Effect of mitoxantrone on LNCaP cells according to the timing of androgen ablation. LNCaP cells were maintained in standard media (SM) for 48 h. The SM was then replaced with charcoal-stripped media (CSM) for 48 h, and the CSM was finally replaced with SM for 48 h. In addition, the cells were treated with various concentrations of mitoxantrone for 48 h before the use of CSM (pre-treatment), at the same period as the use of CSM (simultaneous-treatment) or after the use of CSM (post-treatment). Following 96 h of incubation, cell viability was determined by the in vitro cell growth assay. Each point represents the mean of triplicate analyses with standard deviation. * differs from the other treatment groups ($P < 0.05$) by Student's *t*-test

2-, 1.5- and 4-fold, respectively, above the tumour volume in group 2. As shown in Figure 3B, changes in serum PSA levels almost paralleled changes in tumour volume; that is, by 12 weeks after castration, serum PSA in groups 1, 3 and 4 reached 2-, 2- and 4.5-fold above baseline PSA level before castration, respectively, compared with 20% lower than precastrate baseline in group 2. Furthermore, the TUNEL staining using tumour tissues harvested 4 weeks after castration revealed that apoptotic cell death was extensively induced in LNCaP tumours in group 2 compared to other groups. Apoptotic cells in group 2 detected by the TUNEL staining were 3-, 2- and 9-fold higher than those in group 1, 3 and 4, respectively (Figure 3C).

### Effects of the interval and dosage of mitoxantrone therapy on LNCaP tumour growth

To determine the optimal dosage, male mice bearing LNCaP tumours were injected with a total of 0.5 mg mitoxantrone divided into 2 (i.e. 0.25 mg daily, group 1), 5 (i.e. 0.1 mg daily, group 2) and 10 days (i.e. 0.05 mg daily, group 3) from the day of castration. As shown in Figure 4A, intensive administration of mitoxantrone achieved the most significant antitumour activity. By 12 weeks postcastration, the mean tumour volumes in group 2 and 3 reached 1.5- and 2-fold above the tumour volume in group 1, respectively. Changes in serum PSA levels showed similar pattern to those in tumour volume; that is, by 12 weeks after castration, the mean serum PSA increased 1.2- and 1.5-fold above precastrate levels in group 2 and 3, respectively, compared with remaining 60% lower than precastrate level in group 1 (Figure 4B).

### DISCUSSION

Prostate cancers are considered to be a heterogeneous complex of AD and AI cells, which explains the temporary control of prostate

*© 2001 Cancer Research Campaign*

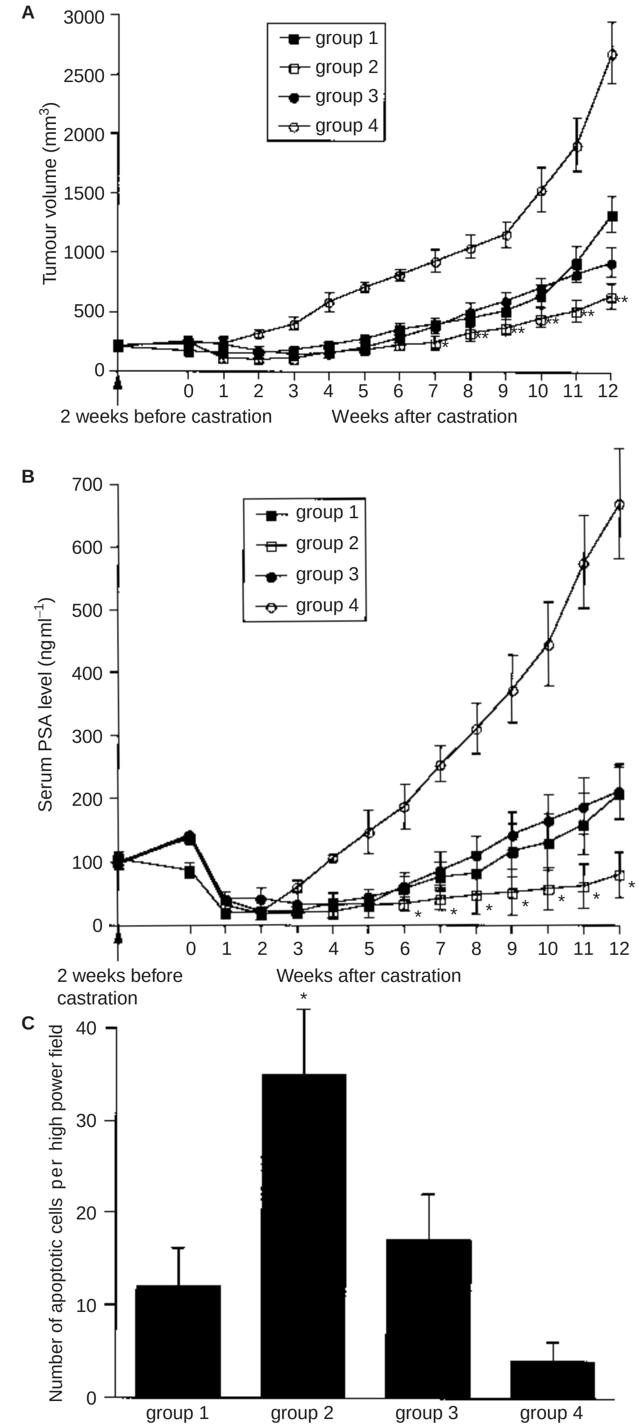

**Figure 3** Effects of mitoxantrone therapy on LNCaP tumour growth according to the timing of castration. (**A**) Mice bearing LNCaP tumours were randomly selected for administration of mitoxantrone from 2 weeks before castration (group 1), the day of castration (group 2), 2 weeks after castration (group 3) or no treatment with mitoxantrone as the control (group 4). Each data point represents the mean tumour volume in each experimental group with standard deviation.** and * differ from the other treatment groups ($P < 0.01$ and $P < 0.05$, respectively) by Student's *t*-test. (**B**) Changes in serum PSA levels in mice injected with the LNCaP cells. Each point represents the mean PSA level in each experimental group with standard deviation.* differs from the other treatment groups ($P < 0.01$) by Student's *t*-test. (**C**) Four weeks after castration, LNCaP tumours were harvested from each treatment group for the detection of apoptosis using TUNEL staining. The number of positively stained cells per high power field in five random fields was counted and averaged. * differs from the other treatment groups ($P < 0.01$) by Student's *t*-test

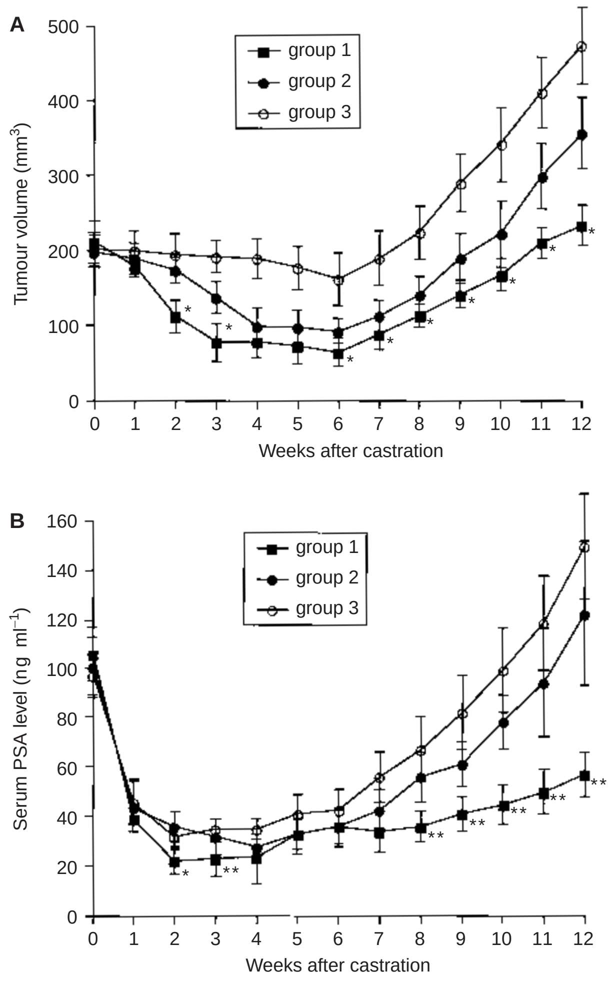

**Figure 4** Effects of mitoxantrone therapy on LNCaP tumour growth according to the timing of castration. (**A**) A total of 0.5 mg mitoxantrone was administered by intravenous injection into each nude mouse bearing LNCap tumour. When tumours reached about 200 mm$^3$ in volume, mice were castrated and randomly selected for administration of 0.25 mg mitoxantrone from the day of castration to 1 day after castration (group 1), 0.1 mg from the day of castration to 4 days after castration (group 2), 0.05 mg from the day of castration to 9 days after castration (group 3). Each data point represents the mean tumour volume in each experiment with standard deviation * differs from the other treatment groups ($P < 0.01$) by Student's *t*-test. (**B**) Changes in serum PSA levels in mice injected with the LNCaP cells. Each point represents the mean PSA level in each experimental group with standard deviation. ** and * differ from the other treatment groups ($P < 0.01$ and $P < 0.05$, respectively) by Student's *t*-test cancer with androgen-withdrawal therapy. A possible explanation for progression to androgen-independence is that continuous suppression of AD cells will ultimately lead to an overgrowth of cells that are not sensitive to androgen ablation (Tang and Porter, 1997). To overcome androgen unresponsiveness, different regimens using both experimental and/or approved chemotherapeutic agents have been explored, however, to date, no chemotherapeutic agent has demonstrated improved survival in patients with advanced prostate cancer (Oh and Kantoff, 1998), emphasizing the need for novel therapeutic approaches to achieve more favourable effects than the current conventional regimen.

Mitoxantrone is an anthracenedione that has demonstrated inhibitory activity in a variety of malignancies, including prostate cancer (Tannock et al, 1996; Kantoff et al, 1999). It is a drug that is well suited for patients with advanced prostate cancer, because it

 *© 2001 Cancer Research Campaign*

causes relatively modest toxicity. In fact, recent phase II reports using mitoxantrone have documented improved response rates in hormone-refractory disease (Hudes et al, 1995; Tannock et al, 1996; Kantoff et al, 1999). Based on these studies, the Food and Drug Administration recently approved mitoxantrone for palliative treatment of hormone-refractory prostate cancer. However, mitoxantrone as a second-line treatment modality has been reported to be of only limited value in improving survival. Therefore, in the present study, using the AD human prostate LNCaP tumour model, we attempted to determine whether it is possible to achieve more favourable effects by directly combining mitoxantrone and androgen-withdrawal therapies.

We first demonstrated that mitoxantrone-induced apoptosis into LNCaP cells is synergistically enhanced by androgen ablation with the use of CSM, and this synergistic effect becomes most remarkable when LNCaP cells were simultaneously treated with mitoxantrone and CSM. Similar to the results of in vitro studies, LNCaP tumour growth in vivo and the increase in serum PSA levels were significantly suppressed by the simultaneous injection of mitoxantrone with castration compared to the administration of mitoxantrone before or after castration. Extensive apoptosis was demonstrated by the TUNEL staining in LNCaP tumours treated with simultaneous castration and mitoxantrone compared to tumours treated with mitoxantrone before or after castration. Furthermore, the second set of in vivo studies revealed that the efficacy of simultaneous mitoxantrone and androgen withdrawal therapy is notable by the administration of mitoxantrone at a higher dosage within shorter interval.

To date, the efficacy of chemotherapy, either as a single agent or in combination therapy, has been traditionally evaluated in patients with hormone-refractory disease (Oh and Kantoff, 1998), and when used in this end-stage setting, none have demonstrated improved survival. A more rational strategy to improve survival by delaying emergence of the AI phenotype would initiate treatment earlier to enhance castration-induced apoptosis rather than the conventional approach of treating patients with established hormone-refractory disease. To achieve more powerful antitumour effect, it would also be important to administer chemotherapeutic agents intensively in a short period. Although the addition of chemotherapeutic agent to androgen ablation for newly-diagnosed prostate cancer has failed to demonstrate improvement of survival and quality of life in the clinical setting (Miyake et al, 1996; de Reijke et al, 1999), our present experimental results suggest the usefulness of intensive mitoxantrone therapy with simultaneous androgen withdrawal as a primary therapy for advanced prostate cancer.

### REFERENCES

Denis L and Murphy GP (1993) Overview of phase III trials on combined androgen treatment in patients with metastatic prostate cancer. *Cancer* **72**: 3888–3895

de Reijke TM, Keuppens FI, Whelan P, Kliment J, Robinson MR, Rea LA, Sylvester RJ and Members of the European Organization for Research in Cancer Therapy Genitourinary Group (1999) Orchiectomy and orchiectomy plus mitomycin C for metastatic prostate cancer in patients with poor prognosis: the final results of a European Organization for Research in Cancer Therapy Genitourinary Group trial. *J Urol* **162**: 1658–1665

Gleave M, Tolcher A, Miyake H, Nelson C, Brown B, Beraldi E and Goldie J (1999) Progression to androgen independence is delayed by adjuvant treatment with antisense Bcl-2 oligodeoxynucleotides after castration in the LNCaP prostate tumor model. *Clin Cancer Res* **5**: 2891–2898

Hudes GR, Nathan FE, Khater C, Greenberg R, Gomella L, Stern C and McAleer C (1995) Paclitaxel plus estramustine in metastatic hormone-refractory prostate cancer. *Semin Oncol* **22**: 41–45

Kantoff PW, Halabi S, Conaway M, Picus J, Kirshner J, Hars V, Trump D, Winer EP and Vogelzang NJ (1999) Hydrocortisone with or without mitoxantrone in men with hormone-refractory prostate cancer: results of the cancer and leukemia group B 9182 study. *J Clin Oncol* **17**: 2506–2513

Miyake H, Hara I, Fujisawa M, Eto H, Okada H, Arakawa S and Kamidono S (1996) Comparison of hormonal therapy and chemohormonal therapy in patients with newly diagnosed clinical stage D prostate cancer. *Int J Urol* **3**: 472–477

Miyake H, Nelson C, Rennie PS and Gleave ME (2000a) Testosterone-repressed prostate message-2 is an antiapoptotic gene involved in progression to androgen independence in prostate cancer. *Cancer Res* **60**: 170–176

Miyake H, Nelson C, Rennie PS and Gleave ME (2000b) Acquisition of chemoresistant phenotype by overexpression of the antiapoptotic gene testosterone-repressed prostate message-2, in prostate cancer xenograft models. *Cancer Res* **60**: 2547–2554

Miyake H, Tolcher A and Gleave ME (2000c) Chemosensitization and delayed androgen-independent recurrence of prostate cancer with the use of antisense Bcl-2 oligodeoxynucleotides. *J Natl Cancer Inst* **92**: 34–41

Oh WK and Kantoff PW (1998) Management of hormone refractory prostate cancer: current standards and future prospects. *J Urol* **160**: 1220–1229

Tang DG and Porter AT (1997) Target to apoptosis: a hopeful weapon for prostate cancer. *Prostate* **32**: 284–293

Tannock IF, Osoba D, Stockler MR, Ernst DS, Neville AJ, Moore MJ, Armitage GR, Wilson JJ, Venner PM, Coppin CM and Murphy KC (1996) Chemotherapy with mitoxantrone plus prednisone or prednisone alone for symptomatic hormone-resistant prostate cancer: a Canadian randomized trial with palliative end points. *J Clin Oncol* **14**: 1756–1762

© 2001 Cancer Research Campaign
